# Development of a Heavy Metal Sensing Boat for Automatic Analysis in Natural Waters Utilizing Anodic Stripping Voltammetry

**DOI:** 10.1021/acsestwater.1c00192

**Published:** 2021-10-20

**Authors:** Qiuyue Yang, Bhawna Nagar, Ruslán Alvarez-Diduk, Marc Balsells, Alessandro Farinelli, Domenico Bloisi, Lorenzo Proia, Carmen Espinosa, Marc Ordeix, Thorsten Knutz, Elisabetta De Vito-Francesco, Roza Allabashi, Arben Merkoçi

**Affiliations:** † Nanobioelectronics and Biosensors Group, Catalan Institute of Nanoscience and Nanotechnology (ICN2), CSIC, and The Barcelona Institute of Science and Technology, Campus UAB, Bellaterra, 08193 Barcelona, Spain; ‡ Universitat Autònoma de Barcelona, Department of Material Science, Campus de la UAB, Plaça Cívica, Bellaterra, 08193 Barcelona, Spain; § École Polytechnique Fédérale de Lausanne (EPFL) Valais Wallis, Laboratory of Physical and Analytical Electrochemistry, Rue de l’Industrie 17, 1950 Sion, Switzerland; ∥ University of Verona, Department of Computer Science, Ca Vignal 2, Strada le Grazie 15, 37134 Verona, Italy; ⊥ Department of Mathematics, Computer Science, and Economics, University of Basilicata, 85100 Potenza, Italy; # BETA Technological Center, University of Vic-Central University of Catalonia (UVic-UCC), 08500 Vic, Spain; @ CERM, Center for the Study of Mediterranean Rivers, University of Vic-Central University of Catalonia (UVic-UCC), 08560 Manlleu, Spain; ∇ Go Systemelektronik GmbH, Falunerweg 1, D-24109 Kiel, Germany; ● University of Natural Resources and Life Sciences, Institute for Sanitary Engineering and Water Pollution Control, Muthgasse 18, 1190 Vienna, Austria; ○ ICREA, Pg. Lluís Companys, 23, Barcelona 08010, Spain

**Keywords:** automatic sampling, automatic sensing system, spatial assessment of heavy metal, screen-printed electrode, square-wave voltammetry

## Abstract

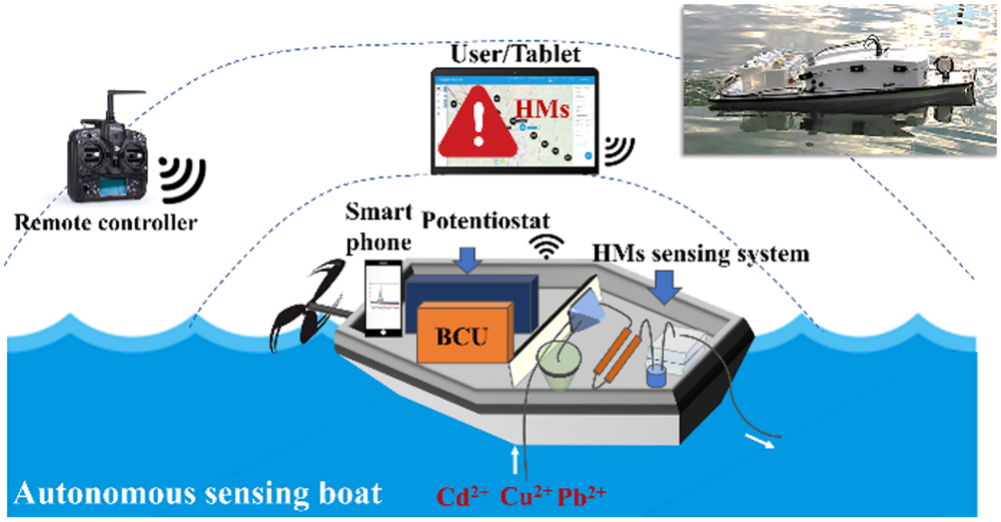

Determination of the levels of heavy metal ions would support assessment of sources and pathways of water pollution. However, traditional spatial assessment by manual sampling and off-site detection in the laboratory is expensive and time-consuming and requires trained personnel. Aiming to fill the gap between on-site automatic approaches and laboratory techniques, we developed an autonomous sensing boat for on-site heavy metal detection using square-wave anodic stripping voltammetry. A fluidic sensing system was developed to integrate into the boat as the critical sensing component and could detect ≤1 μg/L Pb, ≤6 μg/L Cu, and ≤71 μg/L Cd simultaneously in the laboratory. Once its integration was completed, the autonomous sensing boat was tested in the field, demonstrating its ability to distinguish the highest concentration of Pb in an effluent of a galena-enriched mine compared to those at other sites in the stream (Osor Stream, Girona, Spain).

## Abstract 2

An autonomous sensing boat for heavy metal spatial assessment can distinguish the highest concentration of Pb in an effluent of a galena-enriched mine compared with those of other sites in the stream.

## Introduction

Freshwater, a necessity for humans and other life forms, has been continuously compromised by inclusion of heavy metal (HM) pollutants from various natural and anthropogenic processes.^1^ The HM pollutants in water adversely affect humans, animals, and plants due to their tendency to bioaccumulate, their biomagnification, and their environmental persistence.^2^ They pose a serious threat to humans and other living organisms. According to the World Health Organization (WHO) guidelines, Pb, Cd, Cr, and other heavy metals must be controlled in food sources to ensure public safety.^3^ The maximum permitted concentrations of Pb, Cd, and Cu are 10 μg/L, 3 μg/L, and 2 mg/L, respectively.^3^ Monitoring HM pollutants in natural waters below these low concentrations is an urgent need.

Various techniques can determine HMs quantitatively such as atomic absorption spectrometry (AAS),^4^ inductively coupled plasma mass spectrometry (ICP-MS),^5^ inductively coupled plasma atomic emission spectrometry (ICP-AES),^6^ etc. They are highly accurate and quite costly and have complicated sample handling that requires bulky equipment with specialized personnel.

On the contrary, electrochemical techniques are promising due to portability, simplicity, and fast detection.^7,8^ Square-wave anodic stripping voltammetry (SWASV) is one of the most typical techniques for HM detection. Herein, first the HM cations (M^*n*+^) are concentrated and reduced on the electrode surface (to M^0^) by applying a negative potential. Then, they are reoxidized (to M^*n*+^) by applying a reverse potential in square-wave pulses; in the meantime, the current and potential during the reoxidation process are recorded as a voltammogram. Consequently, the HM species and concentration could be known by the peak potential and current intensity, respectively.^9^

On-site HM detection in a body of water, avoiding any pretreatment and maintaining the most original characteristics of HMs, has shown the advantages compared to the traditional approaches for HM distribution assessment in waters involving manual sampling, off-site detection, and possible contamination.^10−15^ To further decrease the labor errors and cost from on-site HM measurements, several automatic sensing probes based on anodic stripping voltammetry have been reported; however, they still suffer from the low portability caused by the large size and lack of remote operation and automation, requiring humans on deck to control the movements of sensing probes between different testing sites, which induces a high cost especially in large lakes and rivers.^16−19^ A fully autonomous sensing tool for on-site HM measurements in natural waters has rarely been reported.

To fill this gap, we developed an autonomous sensing boat with a programmable data collection campaign to assess the spatial distribution of HM pollutants in natural waters. With the aim of automatic sampling and detection, a low-cost fluidic HM sensing system (FSS), as the key sensing component, was fabricated. The autonomous boat was constructed on the basis of the commercial vehicle and adapted for the integration of the FSS and the corresponding electronic controlling unit. Then, the sensing performance with respect to Cd, Pb, and Cu of the FSS was investigated in both deionized water and river water in the laboratory. Finally, the autonomous sensing boat was examined in a mine effluent (in Osor Stream, Girona, Spain).

## Experimental Section

### Reagents and Equipment

Hydrochloric acid (37%, 3203312.5L) and standard heavy metal solutions (Cd, Cu, and Pb at 1000 ppm, AAS grade) were acquired from Sigma-Aldrich. Degassers (bubble trap), tubings (three-stop tygon), and connectors were from Darwin Microfluidics. The peristaltic pump was a model Perimax12 pump from SPETEC. The mini-potentiostat was a model EmStatBlue instrument from Palmsens. The autonomous surface vehicle (Lutra Prop Series, 1.5 m in length) powered by a lithium polymer battery (4 S, 16 Ah, 10 C, 16 V) was acquired from Platypus LLC.^20^ Bluebox (BlueboxT4) was developed by GO Systemelektronik. The software for HM analysis is PStrace 5.8.

### Solutions

Deionized water (18.2 MΩ cm at 25 °C, Milli-Q) was mixed with 37% HCl to prepare HCl dilutions. An individual HM solution was prepared by mixing deionized water with a single HM standard solution (1000 ppm). Mixed HM solutions were prepared with the addition of Cd, Pb, and Cu simultaneously in deionized water.

### Fabrication of SPEs

Screen-printed carbon electrodes (SPE) as a key sensing component were fabricated by a screen printing technique with a DEK248 printer machine (DEK, Weymouth, U.K.).^9^ PET as the substrate was first cleaned with deionized water, ethanol spraying, and nitrogen purging. They were then preheated at 110 °C for 30 min to evaporate solvents and prevent deformation in following steps. The SPEs were fabricated in four steps. (1) Ag paste (C2180423D2 SILVER PASTE-349288, Sun Chemical) was printed to form conductive connections. (2) Then the reference electrode was printed using Ag/AgCl paste (Loctite EDAG AV458, Henkel). (3) The carbon paste was used for patterning working and counter electrodes (C2030519P4 CARBON SENSOR PASTE-267508, Sun Chemical). (4) Finally, the insulating layer was printed (D2070423P5 DIELECT PASTE GRAY, Sun Chemical). The ink was cured at 110 °C for 30 min in an oven after every printing step.

### Square-Wave Anodic Stripping Voltammetry

Detecting HM ions by SWASV included three steps, i.e., deposition, equilibrium, and stripping. During the deposition step, the peristaltic pump was switched on (range of flow rates of 1–7.5 mL/min), and a constant negative potential (deposition potential usually from −1.2 to −1 V) was applied to a working electrode for a deposition period (from 60 to 400 s). Then, during the equilibrium (20 s), the peristaltic pump was stopped. Afterward, the potential was scanned from −1 to 0 V in square-wave pulses in a stripping step with a frequency of 25 Hz, an amplitude of 30 mV, and a potential step of 6 mV.

### DATA Processing

The integral area (*A*) of the HM peak was denoted as the output signal instead of the peak intensity for the peak splitting that occurred when testing mixed heavy metal solutions. The limit of detection (LOD) was defined as 3 times the standard deviation of the minimum concentration with measurable results. Likewise, the limit of quantification (LOQ) was defined as 10 times the standard deviation of the minimum concentration with measurable results.^21^

## Results and Discussion

### Setup of the FSS and the Autonomous Sensing Boat

**Figure 1:**
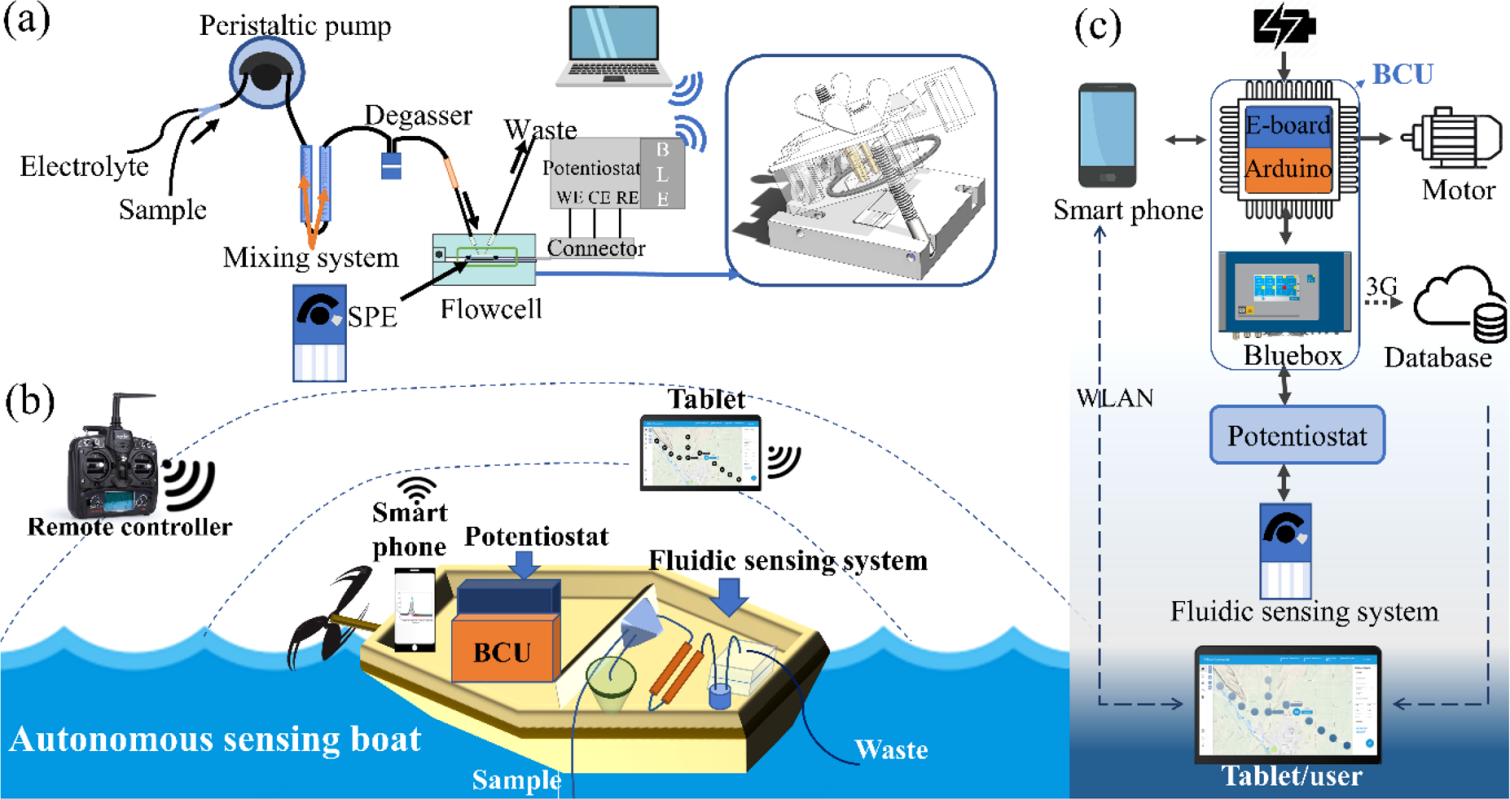
Schematic representation of (a) the FSS, including the sample, prestored supporting electrolyte, peristaltic pump, mixing system, degasser, flow cell (SPE inside), potentiostat, and laptop. The inset shows the open flow cell containing an SPE. (b) Autonomous sensing boat with the FSS extracting in situ water samples and mixing them with a supporting electrolyte in an encapsulated container (green container) in a separate compartment in case of a leakage. (c) Block diagram of the electronic controlling unit describing the architecture and connectivity of the main components.

For automatic, on-site sampling, mixing, and detection, a FSS was developed on the basis of the principle of SWASV, as the key role in the sensing boat. The architecture of FSS is shown in Figure 1a. During measurement, the sample and prestored electrolyte were driven by a peristaltic pump toward the flow cell. The design of two separate inlets and prestored electrolyte was intended for direct and on-site sampling. After mixing and degassing, the mixture was kept on the SPE surface in the flow cell, where the working electrode reduced HM cations in continuous flow during the SWASV deposition step. Afterward, the flow was stopped during equilibrium and stripping, in which the deposited HMs on the working electrode were reoxidized, and the voltammogram was recorded in a portable computer by the potentiostat.

To ensure a homogeneous mixture between the prestored electrolyte and the sample, we created a mixing system consisting of two syringes in series filled with PDMS particles whose diameters were approximately 2 mm (Figure S2b). The performance of this setup is shown in Movie S1, and one can observe how two solutions with different colors (blue and pink) are completely mixed at the end of the process.

Due to the motion generated during the natural movement of the boat when sailing, bubbles that can significantly affect the measurements are produced.^22^ To overcome this effect, a degasser was introduced into the flow system before the electrochemical flow cell. To test the degassing performance, a pink-colored solution, acting as the sample, was mixed intentionally with some air (Movie S2). After flowing out of the degasser, the injected air disappeared, showing the successful removal of all of the air inserted into the system.

A flow cell was customized as a robust electrochemical cell for HM analysis. It was designed on the basis of the fluidic injection system, mainly composed of two pieces of PMMA that encloses the SPE by four screws (Schematic 1a, inset, and Figure S2c,d). When SPE and the O-ring on the cover were compacted, a fixed cell was created (∼100 μL), providing stable conditions for the electrochemical reaction and preventing possible leakage. The embedded electric contacts connected the SPE with the potentiostat (Figure S2d), transmitting the obtained voltammogram to a data analyzer (e.g., smartphone or tablet) by Bluetooth when the analysis had reached completion.

Once assembly and testing of the FSS had reached completion, an autonomous boat (architecture shown in Figure S3) reported in a previous study was used to equip this system.^20^ The boat was originally designed to be commanded by user via a tablet to navigate and monitor indicators in natural waters automatically (e.g., waterline and temperature) through corresponding sensors. It was constructed by adding a Bluebox (sensor control, GO Systemelektronik) to the original boat control unit (BCU) of the commercial vehicle (Lutra series, Platypus), which already includes a smartphone providing GPS data, an E-board for engine control, and operator interface (OI) software in the computer for programing the moving path. However, the proprietary E-board cannot communicate with the added Bluebox directly, interfering with the transmission of data from the sensor to the user. To overcome this, an Arduino Due was integrated on an E-board as the interface with Bluebox. To further accomplish automatic sampling and on-site HM analysis, we transformed this architecture (Figure S3) by adding the developed FSS inside the upgraded one (Figure 1b,c). The mini-potentiostat and peristaltic pump of the FSS were connected to the Bluebox controlling the electrochemical reaction by working potential and sample flow. At the end of the measurements, the obtained voltammograms were analyzed by the Bluebox as the peak potential, current intensity, and integral area, which were stored with a timestamp and the corresponding GPS position in a MYSQL database by 3G technology. For more information about all involved components, see section 3 of the Supporting Information.

In addition to the upgraded hardware, to ensure a direct user interaction with the boat and data transmission, a user-friendly operator interface (OI) is necessary. First (to make it more comfortable during the testing of the campaign in a natural environment), an application of a graphic user interface (GUI), based on the open-source OI of a commercial boat, was transferred to the tablet. The smartphone read remote commands emitted by GUI and transmitted them to the E-board for engine control, so that the GUI allowed users to directly program the data collection campaign (i.e., defining the path that the boat should execute, the speed at which the boat should move, and the time at which to perform the sampling). Simultaneously, the GUI also allowed the users to receive the data from sensors and the status information from the battery via a smartphone and a boat control unit (BCU) even during a mission execution, which strongly assisted operators in regulating the boat behaviors promptly. Moreover, to improve the interaction of the user with the obtained data in the database, a Web application (WAQUIN) and WASCO mobile application were developed to allow the user with a device using a connection to the Internet (e.g., smartphone, tablet, or computer) access in real time, and the HM information on natural waters varying with different time and locations can be even visualized by WAQUIN/WASCO software.

In this way, the user can interact with the sensing boat, program its data collection campaign, and instruct it to drive in the desired area to perform the required measurements. After any campaign, the complete set of data (including calibration data, time, position, and voltammograms) can be transferred from the boat to users for detailed data processing.

### HM Sensing Performance of the FSS and the Automatic Sensing Boat

Compared with Bi-based electrodes, in our study the carbon-based electrodes were selected for its inertness and robustness during deposition and less interference with many heavy metal species (e.g., bismuth-based electrodes can have peak overlapping issues for detecting Cu). To achieve the best sensing performance, key parameters (i.e., supporting electrolyte, concentration, deposition potential, flow time, and flow rate) of the FSS were optimized and are shown in Figures S6–S8. The optimum electrolyte is HCl for supporting the best signal for Cd compared with H_2_SO_4_ and HNO_3_. HCl also has shown better sensing performance with respect to Cd, Pb, and Cu by graphite electrodes reported elsewhere.^23^ The concentration is optimized as 0.05 M, the deposition potential −1.0 V, the flow time 200 s, and the flow rate 3 mL/min. More information is demonstrated in section 5 of the Supporting Information. All optimized parameters were applied in the following measurements.

The FSS was first tested with individual HM standard solutions (Cd, Pb, and Cu) with different concentrations. Figure S9 shows that the FSS has linear responses toward varying HM concentrations in the cases of Cd and Cu (*R*^2^ > 0.90). With regard to Pb, the linearity obtained was not as good as those of Cu and Cd [*R*^2^ = 0.90 (Figure S9b)], which could be caused by the inhomogeneous deposition of Pb onto a carbon paste electrode surface.^24^ The estimated LODs were all at the parts per billion level: 7 μg/L for Cd, 1 μg/L for Pb, and 0.3 μg/L for Cu. The LOQs of Cd, Pb, and Cu were 23, 6, and 1 μg/L, respectively.

To investigate the sensing performance in multi-HM contaminants, the FSS was tested in the standard solutions containing mixed Cd, Pb, and Cu. Compared with individual measurements, as expected, the simultaneous detection did demonstrate the difference in sensitivity and LOD due to the mutual interference. The presence of Pb and Cu decreased the Cd sensitivity dramatically with a LOD of 71 μg/L (Figure S10). It could be attributed to the more negative potential required for Cd deposition compared to Pb and Cu, which induces substance loss on the working electrode during the competition with Pb and Cu.^25,26^ However, Pb and Cu were negligibly affected showing LODs of 1 and 6 μg/L, respectively, both being below the minimum concentration required by WHO guidelines (10 μg/L and 2 mg/L for Pb and Cu, respectively).

**Figure 2:**
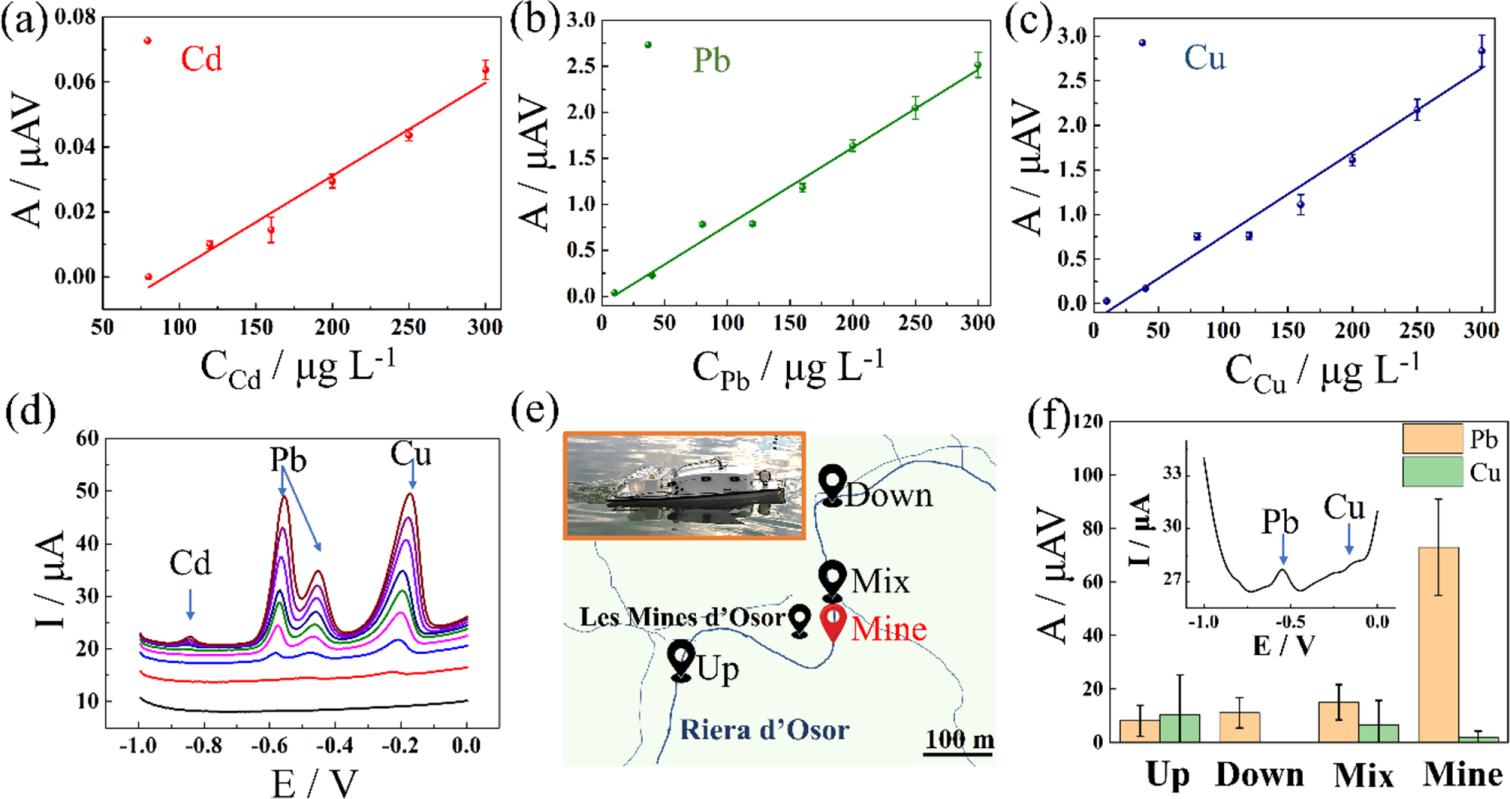
(a–c) Plots of peak area vs concentration in simultaneous measurements for Cd, Pb, and Cu, respectively, in a spiked river sample in the laboratory. (d) Corresponding stripping voltammograms of the simultaneous measurements in a spiked river sample in the laboratory. (e) Schematic illustration of the boat navigating in a campaign experiment. The inset shows a digital photograph of the boat navigating in a river. (f) Determination of Pb and Cu in a campaign experiment. The inset shows a voltammogram obtained in a mine effluent sample, in which a Pb peak centered at −0.5 V can be clearly distinguished.

To further investigate the sensing performance in practicability, the FSS was conducted with real surface water by spiking known concentrations of Cd, Pb, and Cu at the same ratio (Ter River, Vic, Spain) as a standard addition calibration. The FSS demonstrated good linear responses to the varying concentration (Figure 2a–c). The LODs of Cd, Pb, and Cu (99, 0.3, and 3 μg/L, respectively) in river water were at the same level as those in deionized water. It indicates that the impurities in the river have a minimal influence of the river water matrix in the analytical performance of the system. Moreover, the river water matrix did not interfere with identification of HM species as shown by the peaks in the stripping voltammogram (Figure 2d). From left to right, the peaks centered at −0.86, −0.6, and −0.18 V could be easily identified as Cd, Pb, and Cu, respectively. To further investigate if the water matrix influences the accuracy of the fluidic sensing system, the recovery tests were conducted by spiking the standard heavy metals (mixed Cd, Pb, and Cu ions at 180 ppb) with the Ter River water. The recoveries of Cd, Pb, and Cu are 109%, 113%, and 104%, respectively. More details are shown in the Supporting Information.

The robustness of the FSS is crucial for the sensing boat; therefore, the FSS was investigated in terms of repeatability, stability, and reproducibility (shown in Figures S11–S13). In summary, the reproducibilities [relative standard deviation (RSD)] of nine different SPEs toward Cd, Pb, and Cu were 19.57%, 3.86%, and 4.73%, respectively, which are even comparable with those of commercial SPEs (Figure S11). In Figure S12, the repeatabilities toward Cd, Pb, and Cu are acceptable (RSD < 20%) in 28 continuous measurements. Moreover, the FSS also showed good stability within 2 h [RSD < 20% (Figure S13)].

Finally, with the question of how the automatic sensing boat performed in a natural environment, we conducted campaign experiments in a stream affected by the input of an abandoned mine effluent (Osor Stream, Girona, Spain). Figure 2e shows the programmable boat path from the upstream to the downstream in the stream course. Intuitively, the inset of Figure 2e (in the orange square) is the scenario of the boat (approximately 1.5 m in length) navigating under a programmed path automatically, and Movie S3 shows the boat navigating in a larger body of water (Ebro River, Spain).

To further test the HM sensing ability, the automatic sensing boat was instructed to drive into the mine effluent (Figure 2e), which was influenced by the drainage from an enriched-galena Osor mine (Les Mines d’Osor, 41°57′0″ north, 2°35′30″ east). Unfortunately, the on-site analysis was disturbed because the different hydro morphology in the mine effluent (e.g., changing water flow and whirlpool) made the automatic boat consume much more time and power beyond expectation to drive to the tested point that had been set in the program before measurements. The disturbance can be ascribed to the fact that the navigation routine in the GUI was not optimized on the basis of the specific river conditions, and the boat at this stage has low intelligence to respond to sudden uncertainties, which leaves an open challenge for future research. Alternatively, in our study we instructed the sensing boat to collect the samples from the sampling sites (up, mix, mine, and down) and analyzed them by the same sensing boat on shore.

The voltammogram (Figure 2f, inset) of the mine effluent sample showed one clear peak centered at −0.6 V that can be identified as Pb by its peak potential, and a negligible peak at −0.2 V could be attributed to Cu. Figure 2f demonstrates the enhancement in the Pb signal in the mine sample compared to the results from the rest of the stream (up, mix, and down), whereas the Cu signal showed results similar to those of other samples, which indicates Pb with a higher concentration in the mine effluent; however, Cu remained at the concentration in all testing sites. These results were consistent with a previous study of HMs in Mine Osor, in which the concentrations of various HMs were characterized by ICP-MS, and the results showed that in the soil sample, collected from a location (OS-6 in the previous study) adjacent to our testing site, the concentrations of Pb, Zn, and Ba (that originated from a F–Ba–Pb–Zn mine vein) were 2 orders of magnitude higher than those of the rest of the HMs (e.g., Cd and Cu).^28^ Hence, the detected Pb with a higher concentration in mine effluent can be attributed to the drainage or leaching from Mine Osor to surface water, and Cu, not abundant in the mine, remains at the low concentration like the other sites. However, with regard to Cd, it is difficult to detect it not only because the concentration of Cd is relatively low in the mine effluent but also because the high LOD toward Cd is affected by mutual interference with the FSS. Moreover, Zn and Ba as the interferents at high concentrations did not influence the detection without showing any peaks in the voltammogram for their stripping potentials (−1.2 and −2.1 V for Zn and Ba, respectively) were beyond the working potential (from 0 to −1 V).^27−29^

These results suggest the autonomous sensing boat in our study has the ability to analyze the HMs with different concentrations in contaminated water. Compared to the reported studies of an automatic on-site sensing probe based on anodic stripping voltammetry, the autonomous boat has advantages in being fully automated and high portable because of its compact design and small size.^16−19^ Simultaneous detection can be realized with one SPE sensor in the low-cost FSS (cost shown in Tables S1 and S2) without the need for photolithography, which may have a future impact in resource-limited regions.^17−19^

More detailed results about indoor and outdoor validation of this autonomous sensing boat and quantitative analysis are shown in another work, already submitted for publication.^30^

## Conclusion

In this work, an autonomous sensing boat integrated with a FSS was fabricated for HM spatial monitoring in waters based on SWASV. The FSS, as the most important component in the automatic boat, was first tested in the laboratory. The LODs toward Cd, Pb, and Cu were at parts per billion levels in both individual and simultaneous measurements. It was then adapted into an engineered autonomous boat. To investigate the performance, the autonomous sensing boat was tested during a campaign, and it could navigate under a programmable path automatically and distinguish the highest concentration of Pb in the effluent of the galena-enriched mine compared to other sites in the stream. Even though at this stage there are some issues (e.g., the high LOD for Cd caused by mutual interference and possible disturbance by uncertainties in the natural environment as an open challenge for the research community in the future), it is the first autonomous boat operating simultaneous multi-HM detection by anodic stripping voltammetry, which may have a future impact in environmental control. Moreover, compared to the reported on-site and automatic sensing tools, it has the advantages of being small, automated, portable, and cost efficient, which sheds light on HM pollution especially in resource-limited regions.

## Supporting Information Available

Fabrication of SPCE and FSS, architecture of the autonomous boat, optimization of testing conditions, calibrations in single and mixed heavy metal solutions, recovery, reproducibility, and stability tests (PDF)
Video of the mixing system (Movie S1) (AVI)
Video of the degasser expelling bubbles in solution (Movie S2) (AVI)
Video of the autonomous boat navigating automatically in the natural waters (Movie S3) (AVI)

## Supplementary Material

ew1c00192_si_001.pdf

ew1c00192_si_002.avi

ew1c00192_si_003.avi

ew1c00192_si_004.avi
