# Supplementary material for: Development of a Heavy Metal Sensing Boat for Automatic Analysis in Natural Waters Utilizing Anodic Stripping Voltammetry
Source: ACS ES T Water. 2021 Oct 20;1(12):2470–6. doi: 10.1021/acsestwater.1c00192 (PMC8669633; doi:10.1021/acsestwater.1c00192)
Supplement: Supplementary file 1 — ew1c00192_si_001.pdf [file ew1c00192_si_001.pdf]

Development of a heavy metal sensing boat for automatic analysis in natural waters utilizing anodic stripping voltammetry

Qiuyue Yang[a,b ‡], Bhawna Nagar[a,c ‡], Ruslán Alvarez-Diduk[a], Marc Balsells[a], Alessandro Farinelli[d], Domenico Bloisi[d,e], Lorenzo Proia[f], Carmen Espinosa[f,g], Marc Ordeix[f,g], , Thorsten Knutz[h], Elisabetta De Vito-Francesco[i] Roza Allabashi[i] and Arben Merkoçi[a,j,*]

[a]Nanobioelectronics and Biosensors Group, Catalan Institute of Nanoscience and Nanotechnology (ICN2), CSIC and The Barcelona Institute of Science and Technology, Campus UAB, Bellaterra, Barcelona 08193, Spain.

[b]Universitat Autònoma de Barcelona, Department of Material Science, Campus de la UAB, Plaça Cívica, 08193 Bellaterra, Barcelona.

[c]École Polytechnique Fédérale de Lausanne (EPFL) Valais Wallis, Laboratory of Physical and Analytical Electrochemistry, Rue de l'Industrie 17, 1950 Sion, Switzerland.

[d]University of Verona, Department of Computer Science, Ca Vignal 2, Strada le Grazie 15, 37134, Verona, Italy.

[e]Department of Mathematics, Computer Science, and Economics, University of Basilicata, Italy.

[f]BETA Technological Center, University of Vic – Central University of Catalonia (UVic – UCC), Vic, Spain.

[g]CERM, Center for the Study of Mediterranean Rivers, University of Vic – Central University of Catalonia (UVic-UCC), Manlleu, Spain

[h]Go Systemelektronik GmbH; Falunerweg 1, D-24109 Kiel, Germany

[i]University of Natural Resources and Life Sciences, Institute for Sanitary Engineering and Water Pollution Control, Vienna, Muthgasse 18, 1190, Vienna

[j]ICREA, Pg. Lluís Companys, 23, Barcelona 08010, Spain.

Abstract: Determination of heavy metal ions would support assessment of sources and pathways of water pollution. However, traditional spatial assessment by manual sampling and off-site detection in laboratory is expensive, time consuming and requires trained personnel. Aiming to fill the gap between on-site automatic approaches and laboratory techniques, we developed an autonomous sensing boat for on-site heavy metal detection using square-wave anodic stripping voltammetry. A fluidic sensing system was developed to integrate in the boat as the critical sensing component, and was able to detect up to 1 μg/L of Pb, 6 μg/L of Cu and 71 μg/L of Cd simultaneously in laboratory. Once its integration was completed, the autonomous sensing boat was tested in the field, demonstrating its ability to distinguish the highest concentration of Pb in the effluent of a mine compared to other sites in the stream (Osor Stream, Girona, Spain).

1. Fabrication of SPEs by screen printing techniques

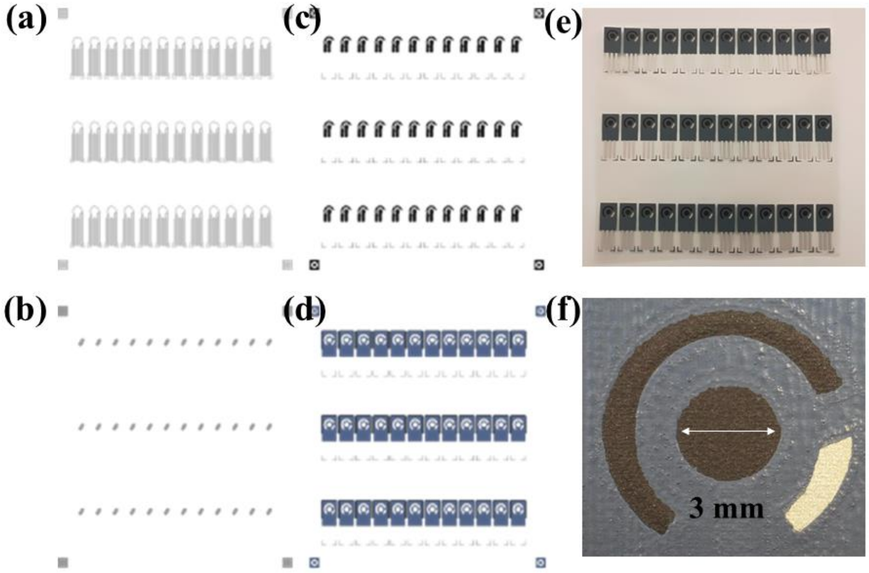

Figure S1 Screen printed electrode (SPE) follows the sequence, different paste and different pattern shown here as (a)first layer Ag pattern, (b) second layer Ag/AgCl pattern, (c) third layer of Carbon paste, and (d)fourth layer of dielectric paste. (e)The photo of one sheet printed sensors including 36 SPEs (f) The photo and dimension of working electrode showing a uniform appearance.

2. Fluidic sensing system

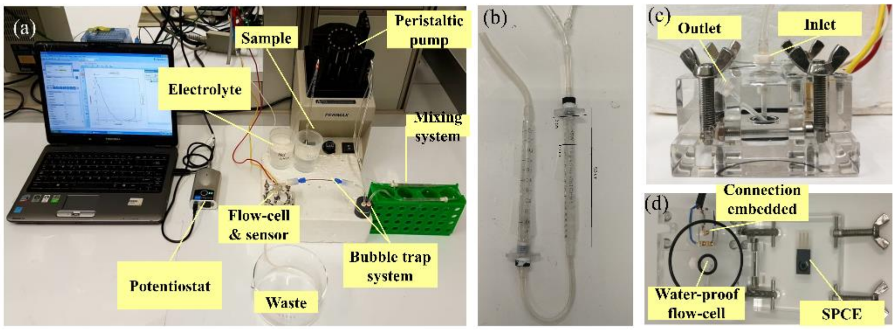

Figure S2. Photo of (a) the fluidic sensing system in laboratory for optimization, individual detection, and simultaneous detection in laboratory, (b)homemade mixers (12 cm*4 cm) including two syringes (d=6 mm) with small PDMS particles filling inside. (c) the encapsulated flowcell with the size of 6cm*6cm*3cm including 4 screws, two pieces of PMMA, one inlet and one outlet, and (d) the open flowcell with two o-rings with diameter of 4.2 cm, and 8mm respectively, which limits the flow and provides a stable chamber (~50 μL) for electrochemical reaction and customized imbedded electronic connection with SPE and mini-potentiostat.

3. Architecture of Autonomous sensing boat and the functions of each component

The INTCATCH architecture of electronic controlling part is divided in 3 main components: Boat Control Unit (BCU) smartphone, and tablet (Figure S3).

BCU is composed of a Bluebox, and a proprietary E-board integrated with an Arduino Due.

Bluebox communicates directly with the sensors and it is responsible for interpreting the electrical signals, converting them to meaningful values, and uploading the data in the Cloud Database for permanent storage via mobile telecommunications technology (i.e., 3G connection). The choice of the Bluebox system as a gateway for all sensors is based on a project requirement and it ensures the possibility to read a wide range of sensors using a unique and well-defined protocol (i.e., serial communication). The use of mobile telecommunications technology for data transfer to the Cloud Database ensures data streaming whenever the mobile network is present without any restriction on the range. Data are also stored locally (i.e., on the Bluebox system) and can be recovered whenever the connection is re-established (e.g., after a data collection campaign has been performed in a remote area).

The orginal/proprietary E-board connects and provides power to all the components of the boat (BlueBox, engines, smartphone, RC receiver). The E-Board is modified by an Arduino Due which reads the sensor values from the Bluebox via serial port and sends them to the smartphone via a custom protocol over USB. The serial protocol was chosen because the serial port is widely available on most computational units and it was already in use by the Bluebox system. The USB interface was chosen because it is easily available on most smartphones. In addition, the E-board receives control commands for the engines from the smartphone and from the Remote controlling (RC) receiver.

The smartphone is the main computational unit of the boat. It controls all the autonomous behaviors of the platform and communicates with the tablet via WiFi to show the sensor values in real-time and to receive commands from the operator (e.g., the path to follow when operating in autonomous mode). The Autonomous Driving Controller module implements the control routines that allows the autonomous boat to follow a specified path while monitoring specific conditions that may trigger the execution of other behavior (e.g., homing when the battery level is critically low). The Data Logger stores the same information that are recorded by the Bluebox system and it is used as a redundant storage repository to backup the sensor data. Data can be accessed by downloading a file from the smartphone and such file can then be uploaded to the Cloud Database.

The tablet is an interface for the operator to control the boat, it displays the boat status (e.g., position over the map) and sensor values and allows the user to manage the data collecting mission. A key function of the tablet concerns the creation of different types of paths by specifying waypoints on the map. Such waypoints are then used by the Waypoint Path Generator module to create a path which is then followed by the boat. The Autonomous Driving Controller allows the user to interact with the boat while the autonomy is in operation. For example, it allows to monitor the status of mission execution (e.g., displaying the boat's position along the path) and to change the parameters for mission execution (e.g., the boat speed).

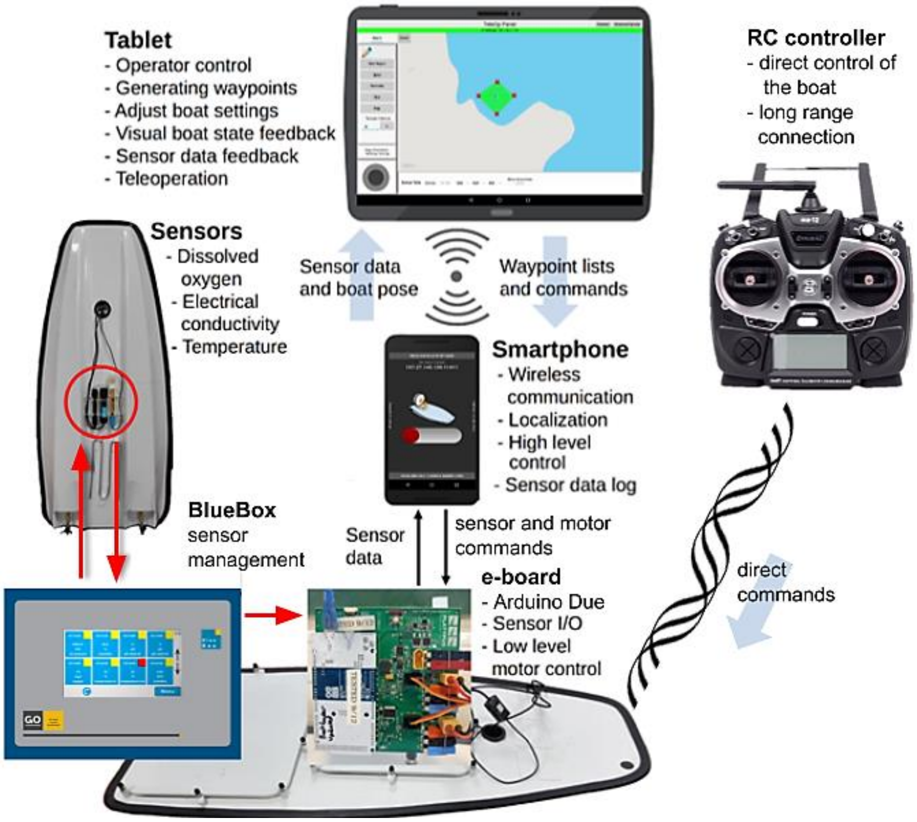

Figure S3: Architecture of the boat control system showing options for manual RC control and autonomous operation through the Control App running on the tablet.

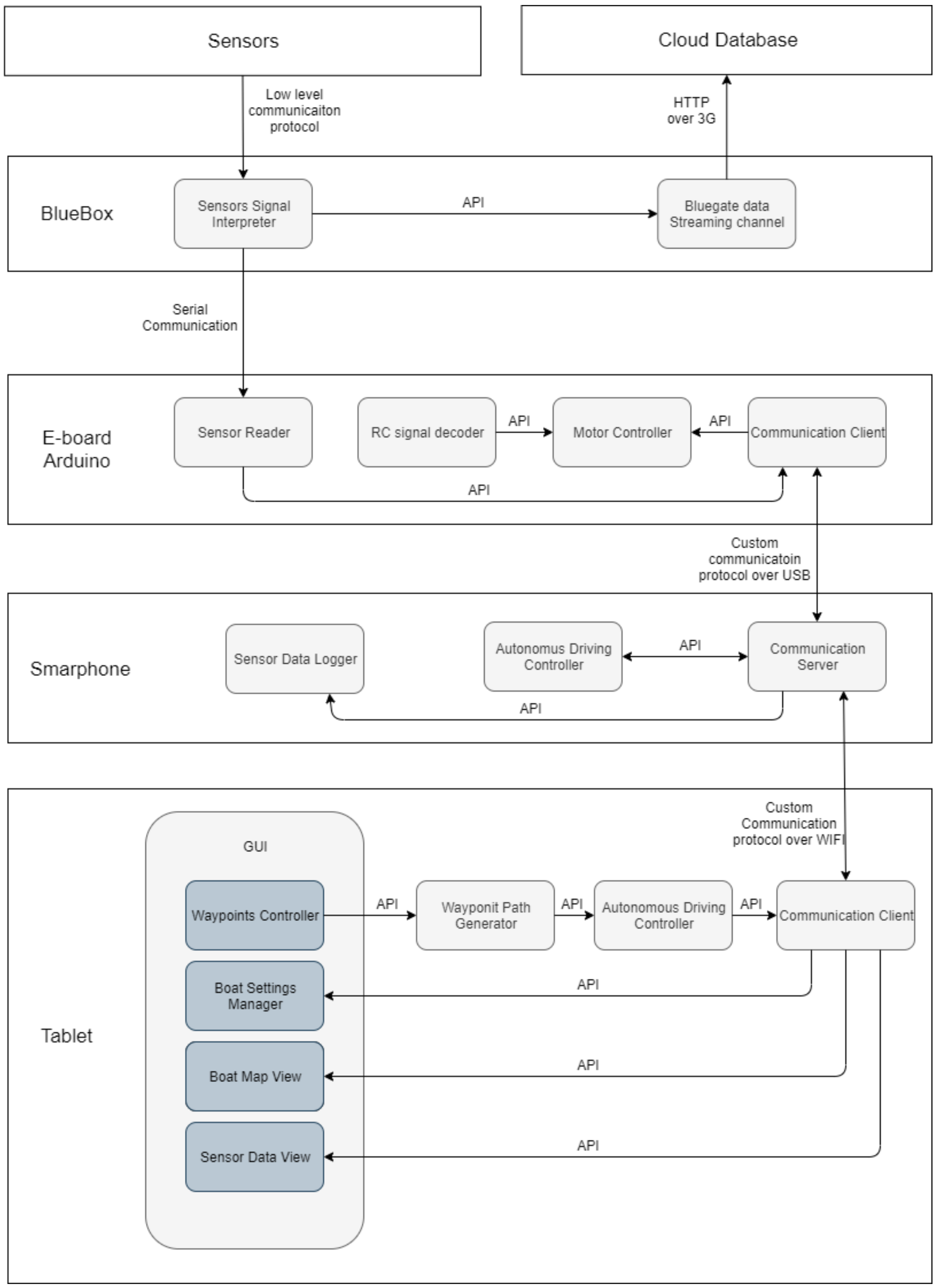

Figure S4: Architecture of the boat software, rectangles with sharp corners represents hardware components, rectangles with rounded corners are software components, arrows describe the communication.

4. Validation of suitcase

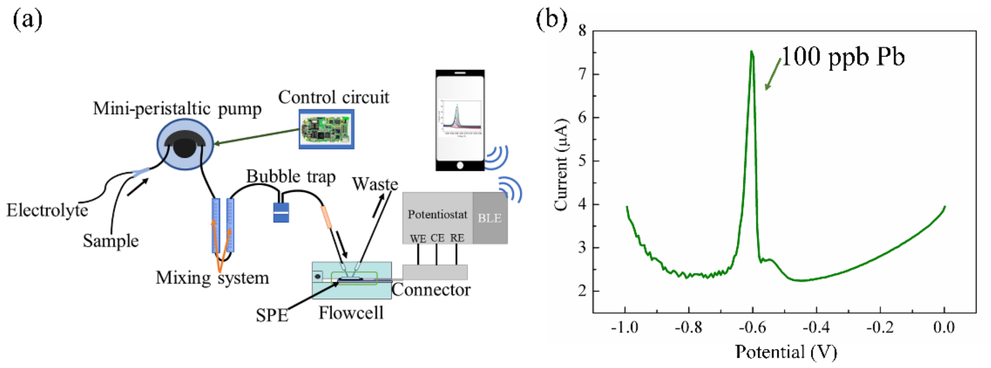

Figure S5. The fluidic system can be integrated into a suitcase container to enhance its portability. The sensing suitcase was tested in 100 ppb Pb solution. (a)The photo of the sensing suitcase. (b)The voltammogram of Pb with all the optimized parameters of 0.05M HCl as supporting electrolyte, 3 ml/min as flowrate, 200s as deposition time and -1 V as deposition potential. The integral area is 0.245 μAV which can match the results of the original fluidic system (0.278 μAV).

5. Optimization

5.1 Selection of supporting electrolyte and optimization of concentration

The supporting electrolytes play important roles in HM detection. They were optimized by detecting Cd (80 μg/L) using HNO_3 (0.05 M), HCl (0.05 M), and H_2SO_4 (0.025 M). Cd rather than Pb or Cu was chosen because Cd^{2+} is considered more sensitive to the influence of acids due to its reduction potential closer to that of H^+.[1] The highest signal was obtained using HCl in Figure S6a. Therefore, HCl was chosen as the supporting electrolyte. The concentration of acid affects HM ions hydrolysis which is critical for deposition and stripping processes. The sensing system was tested using 80 μg/L Cd with different concentrations of HCl. More concentrated HCl causes more free ions which is beneficial for detection until saturation (Figure S3b). The concentration of HCl would also influence the simultaneous HM detection. To study it, mixed HM ions of 20 μg/L Cu, 20 μg/L Pb and 80 μg/L Cd in different concentrations of HCl (0.01M-0.20M) were detected. As shown in Figure S6c, 0.05M of HCl supported the best signals of Pb and Cu with obvious separation between their peaks and a detectable signal of Cd (Figure S6d). Thus, 0.05M of HCl was applied as supporting electrolyte.

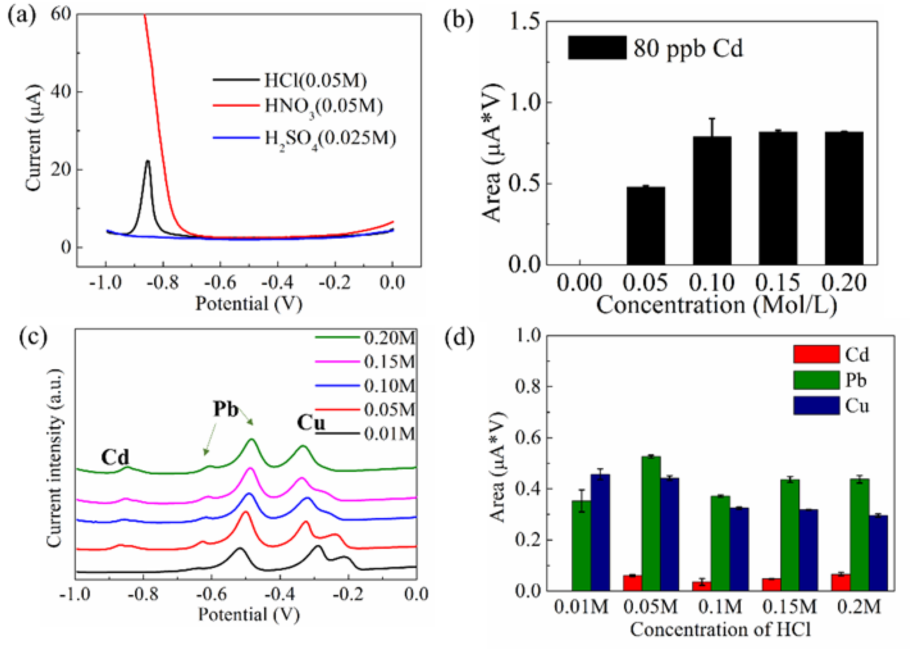

Figure S6. (a)Voltammograms and (b)diagram of integral peak area of 80ppb Cd in different supporting electrolytes. (c) Voltammograms and (d) diagrams of Cd, Pb and Cu in mixed solution with 80ppb Cd, 20ppb Cu and 20 ppb Cu in different concentration of HCl.

5.2 Optimization of flowrate and flowtime

There is a general agreement between the increase of flow rate/time and the increase of sensing signals. However, the limited volume of supporting electrolyte during on-site measurements would not support high flow rate or long flow time. To investigate the optimum flow conditions, Cd at a concentration of 80 μg/L was detected using different flow rates. The sensing signal increased non-linearly with increasing flow rate from 0 to 7.5 mL/min. An optimized flow rate of 3 mL/min was kept for all further measurements due to the following reasons (1) as it shows good Cd sensing signals with the smallest standard deviation and further

increase of flow rate does not show a linear response and (2) the robotic boat has limited space unable to be equipped with high-volume supporting electrolyte. Hence, 3 ml/min was chosen as the optimized flow rate. Similarly, different flow (deposition) time from 60 to 400s for the detection of Cd (80 µg/L) were tested. The results showed a linear relationship between the deposition time and sensing signals. The deposition time of 200s was selected as it showed sufficiently obvious signals with the smallest standard deviation. Moreover, it is a good compromise considering limited analysis time on the robotic boat. For example, the deposition time of 200s handles a maximum of 30 measurements in 2 hours including the pre-calibration and on-site measurements for 5 different tested spots (n=3).

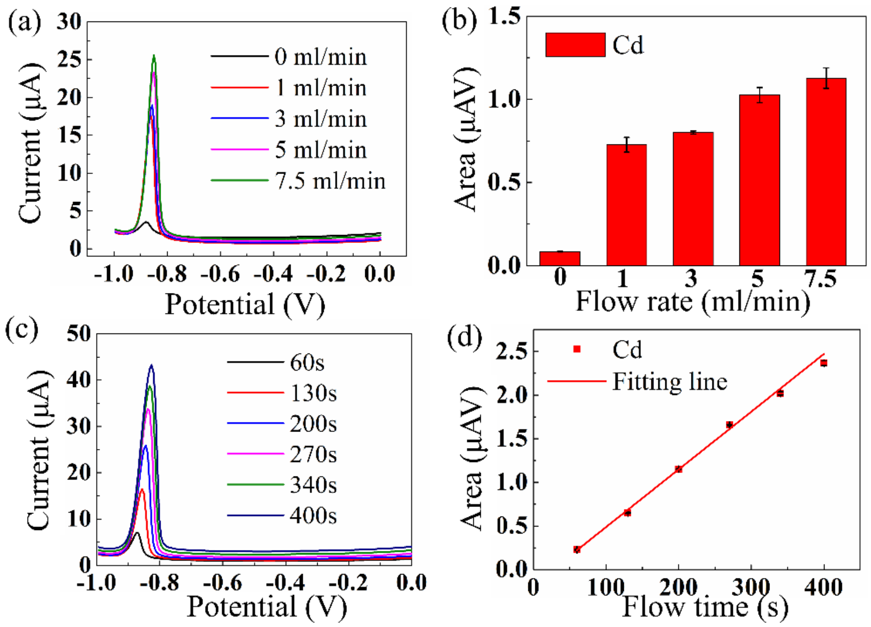

Figure S7 (a) Stripping voltammograms and (b) dia-grams towards detecting 80 ug/L Cd with different flow rate. (c) Stripping voltammograms and (d) dia-grams towards detecting 80 ug/L Cd with different flow time.

5.3 Deposition potential

Applying more negative deposition potential increased the sensing signals but generated bubbles on the working electrode due to hydrogen evolution reaction.[2] They caused damage to the working surface and irreproducible signals. As our observation, the potential equal and larger than -1.1 V facilitated bubble generation on the SPE within the flowcell. Compromising the robustness of the whole system, the more negative potentials were not applied in the following studies. Cd was undetectable below -0.8 V. Hence, a deposition potential of -1.0 V was chosen.

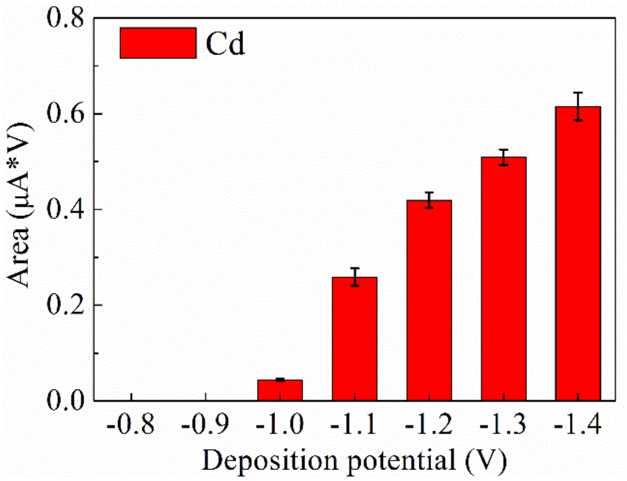

Figure S8 Diagram of Cd in mixed solution of 80ppb Cd, 20ppb Cu and 20 ppb Cu with different deposition potential.

6 Calibration in individual HM solution

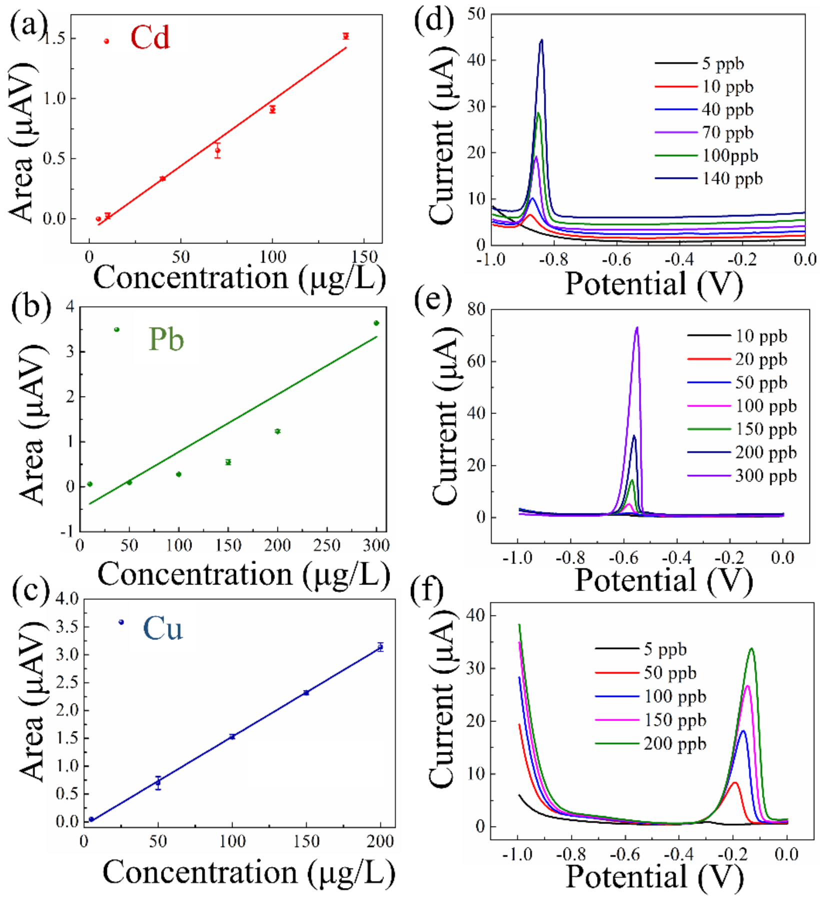

Figure S9. The calibration lines and stripping voltammograms of Cd (a, d), Pb (b,e) and Cu (c,f), respectively in individual HM detection. The fitting lines of Cd, Pb and Cu are $y_{Area}=0.010x_{Conc.}-0.10$ ($R^2=0.98$), $y_{Area}=0.013x_{Conc.}-0.50$ ($R^2=0.91$), and $y_{Area}=0.016x_{Conc.}-0.063$ ($R^2=0.99$), respectively.

7 Simultaneous calibration in mixed solution of Cd, Cu and Pb

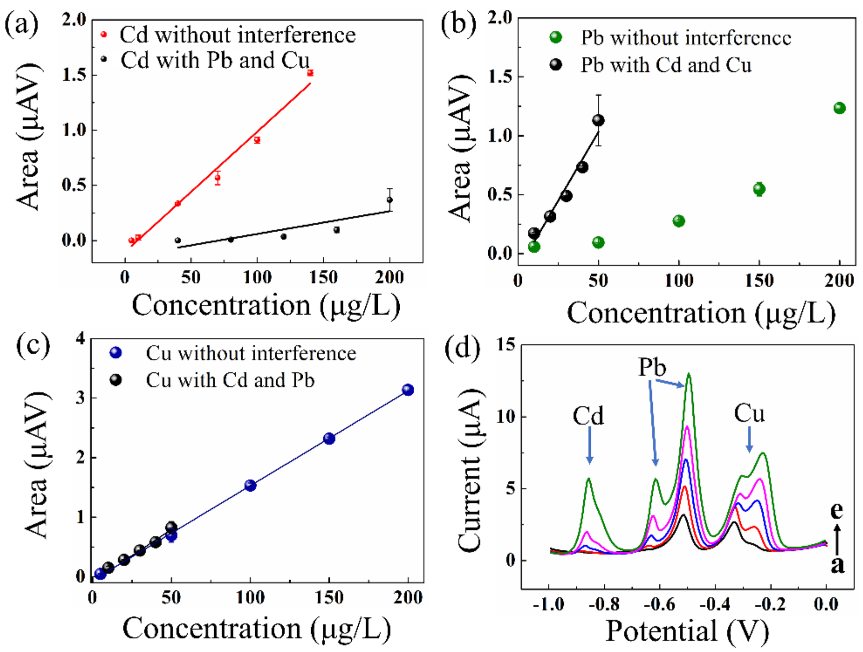

Figure S10. The calibration of (a) Cd (b) Pb and (c) Cu with and without the other two HMs as interferents. (d) The stripping voltammograms are of Cd, Pb and Cu: a. Cd of 40 μg/L, Pb of 10 μg/L and Cu of 10 μg/L; b. Cd of 80 μg/L, Pb of 20 μg/L and Cu of 20 μg/L; c. Cd of 120 μg/L, Pb of 30 μg/L and Cu of 30 μg/L; d. Cd of 160 μg/L, Pb of 40 μg/L and Cu of 40 μg/L; e. Cd of 200 μg/L, Pb of 50 μg/L and Cu of 50 μg/L.

8 Recovery test

Table S1. The accuracy of spiked 180 ppb Pb, Cd, and Cu with Ter River matrix by the fluidic sensing system.

	Calibration Equation	R^2	Tested signals (μAV) (Y)	Calculated Conc.(ppb) (X)	Spiking Conc.(ppb)	Accuracy
Cd	Y=0.00028516X-0.02553	0.9298	0.0308±0.0044	197.5	180	109%
Pb	Y=0.0085X-0.76	0.9843	0.978±0.019	204.4	180	113%
Cu	Y=0.0094X-0.19	0.9693	1.58±0.13	188.3	180	104%

* The sensing range of Cd here was used as 120-300 ppb to reach better R^2(>0.90).

9 Reproducibility of SPE

Nine SPEs from nine sheets of single batch were tested in the mixed solution containing 80 μg/L of Cd, 20 μg/L of Pb and Cu. The relative standard deviation (RSD) for Cd, Pb and Cu was 19.5%, 3.7% and 4.7% respectively (Figure S11a-c). In terms of commercial SPEs, the calculated RSD of Cd, Pb and Cu was 12.6%, 9.5% and 5.5% respectively. There were some failures during detecting Cd by CEs (Figure S11d), which was caused by the different layouts of the customized connector and commercial products.

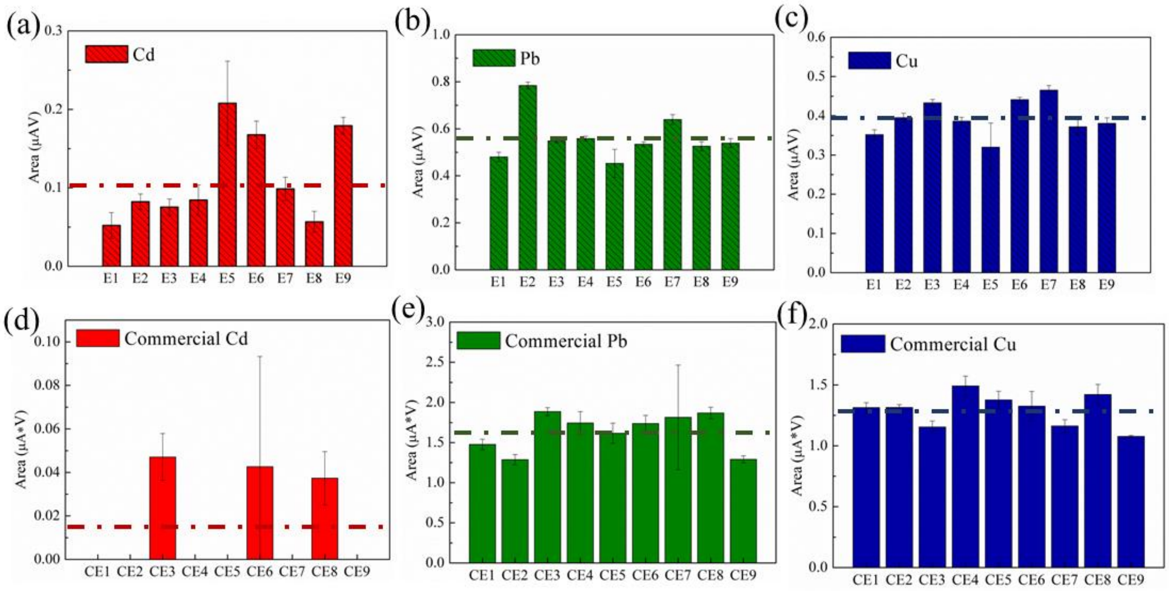

Figure S11. In the mixed solution of 80 ppb Cd, 20 ppb Pb and 20 ppb Cu, the integrated area of Cd from the electrodes from different sheets(a), the integrated area of Pb of the electrodes from different sheets(b), the integrated area of Cu of the electrodes from different sheets(c), sensing performance of two electrodes from one sheet(d), the integrated area of Cd by commercial electrodes(e), the integrated area of Pb and Cu by commercial electrodes.

10 Repeatability

One SPE was tested 30 times in the mixed solution containing Cd, Pb and Cu at the concentration of 80 μg/L, 20 μg/L and 20 μg/L. Figure S12 shows the sensing signals of 30 measurements in 2 hours. The RSD of Cd, Pb and Cu is 35.7%, 5.3% and 1.9%, respectively. The sensing system shows good repeatability for detecting Pb and Cu during all the 30 measurements. On the other hand, the RSD of Cd is relatively high, which originates from the extremely high signals of the first two measurements. It may be caused by the more active sites for Cd in virginal SPEs. By excluding these abnormal signals, the RSD of Cd can be reduced to 18.1%. Thus, it is recommended to pre-test SPEs in HM solutions several times before real applications.

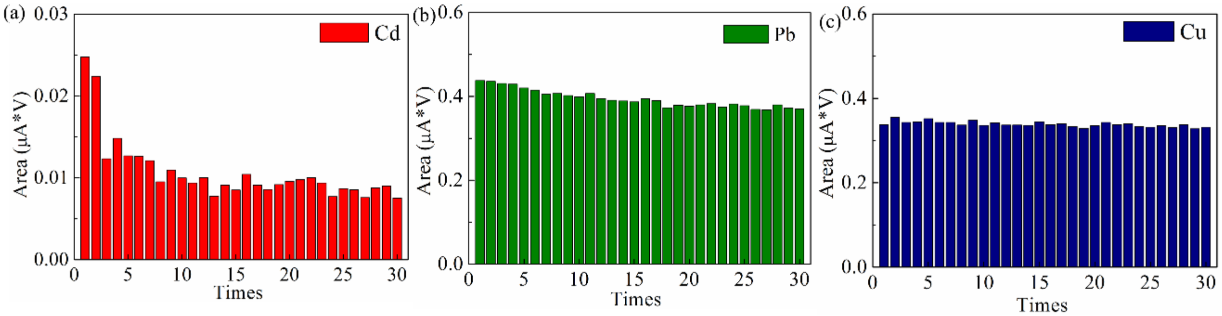

Figure S12. One sensor repeated the measurement 30 times in the mixed solution of 80 ppb Cd, 20 ppb Pb and 20 ppb Cu. The total time is within 2 hours.

11 Stability

The measurements were operated in the mixed solution of Cd, Pb and Cu every 20 min in 8 hours. The sensing system exhibited high stability for Pb and Cu with small RSDs of 6.3% and 7.4% respectively in the whole 8 hours. Regarding to Cd, the RSD in 8 hours (41.71%) was much enhanced compared with that in the first two hours (13.62%). The reason is not clear yet. Generally, the pre-sent results showed the sensing system has good stability (RSD<20%) in the first two hours.

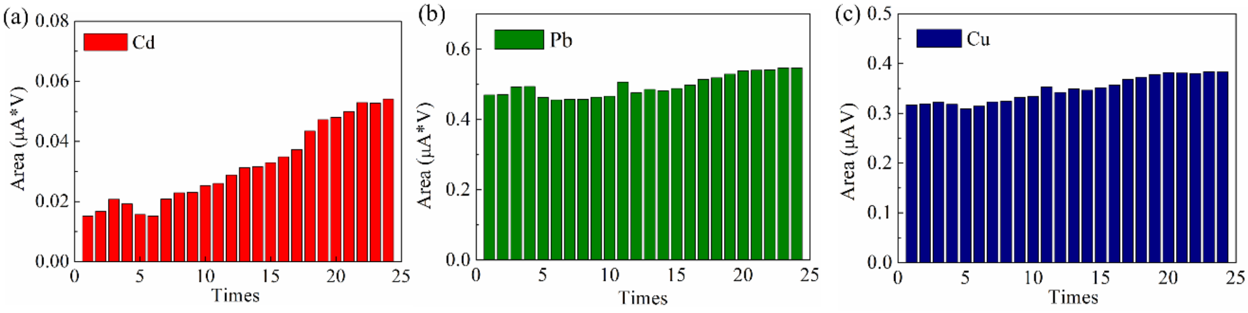

Figure S13. Detection in the mixed solution of 80 ppb Cd, 20 ppb Pb and 20 ppb Cu in 8 hours. Internal time is 20 min, and total number of measurements is 24 times.

12 Cost of fluidic sensing system

Table S2. The material cost summary of SPEs

	unit price (€/g)	Cost (g/sheet)	Cost (€/sheet)
Ag ink	0.859	0.85	0.73
Ag/AgCl ink	2	0.85	1.7
Carbon paste	0.189	1	0.189
Dielectric ink	0.196	0.75	0.147
Substrate	-	-	3.13
Total (€/sheet 36 SPEs)			5.89
Total (€/single SPE)			0.16

Table S3. The material cost summary of the fluidic system

	Materials Cost (€/unit)
minipotentiostat	1850
flowcell	375
degasser	132
mixers	0.48
tubings	14.47
Pump	29.51
SPE	0.16
Total	2401.62

Reference:

1. Zhai, Z.; Huang, N.; Zhuang, H.; Liu, L.; Yang, B.; Wang, C.; Gai, Z.; Guo, F.; Li, Z.; Jiang, X. A Diamond/Graphite Nanoplatelets Electrode for Anodic Stripping Voltammetric Trace Determination of Zn(II), Cd(II), Pb(II) and Cu(II). Appl. Surf. Sci. 2018. DOI: 10.1016/j.apsusc.2018.06.266.
2. Bondue, C. J.; Graf, M.; Goyal, A.; Koper, M. T. M. Suppression of Hydrogen Evolution in Acidic Electrolytes by Electrochemical CO2 Reduction. J. Am. Chem. Soc. 2021. DOI: 10.1021/jacs.0c10397.
